# Intensity-modulated radiotherapy with simultaneous modulated accelerated boost technique and chemotherapy in patients with nasopharyngeal carcinoma

**DOI:** 10.1186/1471-2407-13-318

**Published:** 2013-07-01

**Authors:** Muhammad M Fareed, Abdullah S AlAmro, Yasser Bayoumi, Mutahir A Tunio, Abdul S Ismail, Rashad Akasha, Mohamed Mubasher, Mushabbab Al Asiri

**Affiliations:** 1 Department of Radiation Oncology, King Fahad Medical City, Riyadh, Saudi Arabia; 2 Department of Radiation Oncology, NCI, Cairo University, Cairo, Egypt; 3 Department of Clinical Oncology, University of Alexandria, Alexandria, Egypt; 4 Department of Clinical Research and Biostatistics, King Fahad Medical City, Riyadh, Saudi Arabia

**Keywords:** Intensity-modulated Radiotherapy (IMRT), Simultaneous Modulated Accelerated Radiotherapy (SMART) Boost Technique, Nasopharyngeal Carcinoma

## Abstract

**Background:**

To present our experience of intensity-modulated radiotherapy (IMRT) with simultaneous modulated accelerated radiotherapy (SMART) boost technique in patients with nasopharyngeal carcinoma (NPC).

**Methods:**

Sixty eight patients of NPC were treated between April 2006 and December 2011 including 45 males and 23 females with mean age of 46 (range 15–78). Stage distribution was; stage I 3, stage II 7, stage III 26 and stage IV 32. Among 45 (66.2%) evaluated patients for presence of Epstein-Barr virus (EBV), 40 (88.8%) were positive for EBV. Median radiation doses delivered to gross tumor volume (GTV) and positive neck nodes were 66–70 Gy, 63 Gy to clinical target volume (CTV) and 50.4 Gy to clinically negative neck. In addition 56 (82.4%) patients with bulky tumors (T4/N2+) received neoadjuvant chemotherapy 2–3 cycles (Cisplatin/Docetaxel or Cisplatin/Epirubicin or Cisplatin/5 Flourouracil). Concurrent chemotherapy with radiation was weekly Cisplatin 40 mg/m^2^ (40 patients) or Cisplatin 100 mg/m^2^ (28 patients).

**Results:**

With a median follow up of 20 months (range 3–43), one patient developed local recurrence, two experienced regional recurrences and distant failure was seen in 3 patients. Estimated 3 year disease free survival (DFS) was 94%. Three year DFS for patients with EBV was 100% as compared to 60% without EBV (p = 0.0009). Three year DFS for patients with undifferentiated histology was 98% as compared to 82% with other histologies (p = 0.02). Acute grade 3 toxicity was seen as 21 (30.9%) having G-III mucositis and 6 (8.8%) with G-III skin reactions. Late toxicity was minimal and loss of taste was seen in 3 patients (7.5%) at time of analysis.

**Conclusions:**

IMRT with SMART in combination with chemotherapy is feasible and effective in terms of both the clinical response and safety profile. EBV, histopathology and nodal involvement were found important prognostic factors for locoregional recurrence.

## Background

Radiation therapy is considered as the mainstay of treatment in nasopharyngeal carcinoma (NPC) management. Advances in techniques of radiation delivery, incorporation of chemotherapy and better imaging tools have made it possible to achieve local control rate up to 95%. Radiation Therapy Oncology Group (RTOG) phase II trial 0225 established the feasibility of translating IMRT with or without chemotherapy in NPC patients [1]. IMRT is capable of producing highly conformal dose distributions by manipulating the beam intensity within different parts of each radiotherapy beam. It provides improved tumor coverage and spares critical structures around the tumor (brainstem, parotid glands and optic structures) when compared with conventional and three dimensional conformal therapy techniques for NPC [2,3]. IMRT for NPC decreases late neurologic sequelae and permanent xerostomia by sparing critical portions of the brain stem and the parotid glands respectively [4].

The role of chemotherapy either in neoadjuvant setting before definitive treatment or concurrent with radiation is a matter of great interest. Concurrent chemotherapy with radiation therapy has been shown to improve local control, disease-free survival and overall survival rates for NPC patients with T2 - T4 diseases, or with neck lymphadenopathy. IMRT following neoadjuvant chemotherapy is a strategy that deserves to be optimized and needs to be tested in prospective randomized phase III trials in patients with locoregionally advanced NPC. Neoadjuvant chemotherapy is an effective way to control subclinical metastatic foci, especially in lymph node positive patients of NPC. Moreover, in some patients with large tumors infiltrating the brain stem, it is often difficult to deliver the total required dose to the clinical target volume (CTV) with preservation of critical tissues. Neoadjuvant chemotherapy is often able to provide objective responses in tumor lesions, which offers the possibility to shrink the CTV and reduce toxicity [5,6].

IMRT with simultaneous modulated accelerated radiotherapy (SMART) boost technique to enhance biologically equivalent dose (BED) along with concurrent chemotherapy, although less studied, has shown excellent local control rates with minimal toxicity profile, albeit no further improvement was noted in overall survival [7].

We aimed to evaluate clinical outcomes, efficacy, toxicity profile and associated prognostic factors influencing locoregional and distant control in NPC patients treated with neoadjuvant chemotherapy followed by IMRT and SMART concurrent chemoradiation.

## Methods

### Patients and pretreatment criteria

After institutional review board (IRB) approval, retrospective data of sixty- eight consecutive patients with histologically proven, non-metastatic NPC treated at our department from April 2006 to December 2011 was collected. Pretreatment evaluation consisted of complete history and physical examination, rigid nasendoscopy, magnetic resonance imaging (MRI), computed tomography (CT) imaging of head and neck, CT chest, complete blood count, liver function tests, renal profile, dental and audiological assessment prior to the treatment. After May 2008, all the patients were tested for Epstein-Barr virus (EBV) on histological specimens. Positron emission tomography (PET) imaging, CT abdomen and bone scan were optional and were performed when clinically indicated. Patients who had evidence of distant metastasis were not eligible for this treatment protocol. All patients were treated after taking informed consent. AJCC 2002 cancer staging classification was used to stage tumors.

### Radiation therapy techniques

**Figure 1:**
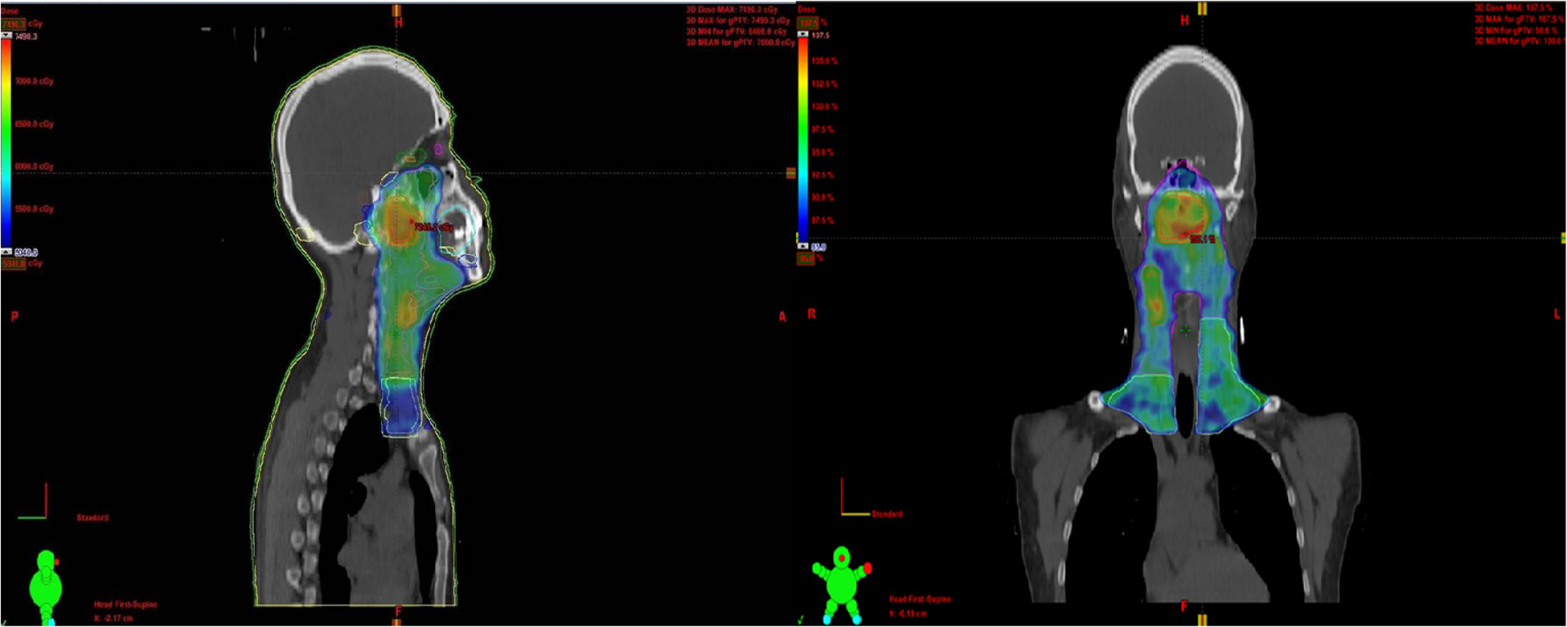
High, intermediate and low dose areas in IMRT – SMART plan for nasopharyngeal carcinoma.

CT simulation was done for all patients in supine position with thermoplastic masks (S-frame) for immobilization. Images were acquired with and without contrast using 3mm slices for planning purpose. Pre-chemotherapy MRI and CT imaging were fused with CT simulation data using co-registration software where deemed appropriate to delineate the tumor. The gross tumor volume (GTV) included all primary and nodal disease seen on MRI and/or CT scan. CTV denoted the subclinical regions at risk for involvement. The high-risk clinical tumor volume (CTV-1) included GTV plus 5–10 mm margin. CTV-2 as designed for potentially involved regions included the nasopharyngeal cavity (limited only to the posterior part of nasal cavity), maxillary sinus (limited to 5-mm anterior to the posterior nasal aperture and maxillary mucosa), pterygopalatine fossa, posterior ethmoid sinus, parapharyngeal space, skull base, anterior third of clivus and cervical vertebra, inferior sphenoid sinus and cavernous sinus, and included the retropharyngeal lymph nodal regions from the base of skull to cranial edge of the second cervical vertebra. The CTV of the neck nodal regions included level II, III, IV, V, which was outlined according to the recommendation by the RTOG/EORTC CTV delineation protocol for head and neck malignancies. The planning target volume (PTV) was created based on each volume with an additional 3-mm margin, allowing for setup variability. Critical normal structures including the brainstem, spinal cord, parotid glands, optic nerves, chiasm, lens, eyeballs, larynx, esophagus, temporomandibular joints, mandible and cochlea were contoured and set as organs at risk (OARs) during optimization. RT was delivered by using a simultaneous-integrated IMRT boost technique. The radiation dose delivered was 66-70 Gy in 33–35 fractions (2 Gy per day) to gross tumor (prechemotherapy volume), 63 Gy in 35 fractions (1.8 Gy per day) to high risk volume and 50.4 Gy in 28 fractions (1.8 Gy per day) to low risk volume (Figure 1).

### Chemotherapy

Fifty six patients (82.4%) with bulky tumors (T4 or N+) received neoadjuvant chemotherapy 2–4 cycles. Regimen used were as; (a) Cisplatin and Docetaxel in 30 patients, (b) Cisplatin and Epirubicin in 16 patients and (c) Cisplatin and 5-Flourouracil in 10 patients. Sixty four patients (91.2%) received concurrent chemoradiation. Chemotherapy used was either weekly Cisplatin 40 mg/m^2^ (40 patients) or 3 weekly Cisplatin 100 mg/m^2^ (24 patients).

### Follow-up

All patients were evaluated weekly during radiation therapy. First follow up was at 6 weeks after the completion of their treatment, then every 2–3 months in the first 2 years, every 6 months from year 2 through year 5, and annually thereafter. Each follow-up included a complete history, physical examination and rigid nasendoscopy. CT scan of the head and neck was performed at 2–3 months post treatment and afterwards as needed. Acute toxicity profile during treatment and first three months was assessed by Common Toxicity Criteria CTC version 3.0 and late toxicities were scored according to the RTOG radiation morbidity scoring criteria at each follow-up.

### Statistical analyses

The actuarial local/regional control, response rates and disease-free survival were calculated by life test method. The duration of time to locoregional failure and distant metastasis was measured from the date of the completion of radiation therapy until documented treatment failure. The duration of DFS was calculated from diagnosis until development of documented pathologic or radiologic recurrence. Log-rank test and chi-square were used to detect the significant difference in survivals between different prognostic groups. Multivariate analysis using the Cox proportional hazard model was performed for the aforementioned endpoints to define independent predictors among various potential prognostic factors.

## Results

Patients’ characteristics are shown in Table 1. Majority of the patients had locally advanced NPC; stage IV (51.4%) and stage III (35%) and majority of cohort had undifferentiated histopathology. Median follow up was of 20 months (range 3–43).

**Table 1:** Patients’ characteristics

**Variables**	**Number (%)**
**Age (years)**	Median 46 (range:15–78)
**Sex**	
Males	45 (66%)
Females	23 (44%)
**T stage**	
T1	13 (19%)
T2	18 (26%)
T3	11 (16%)
T4	26 (38%)
**N stage**	
N0	11 (16%)
N1	22 (32%)
N2	27 (40%)
N3	8 (12%)
**AJCC staging**	
I	3 (4%)
II	7 (10%)
III	26 (38%)
IV	32 (47%)
**Histopathology**	
Undifferentiated	51 (76%)
Poorly differentiated	17 (16%)
unknown	6 (8%)
**EBV positivity**	
Yes	41(60%)
No	5 (8%)
Unknown	22 (32)

Abbreviations: *T* tumor, N nodes, *AJCC* American Joint Commission for Cancer, *EBV* Epstein-Barr Virus.

### Locoregional, distant control and overall survival rates

**Figure 2:**
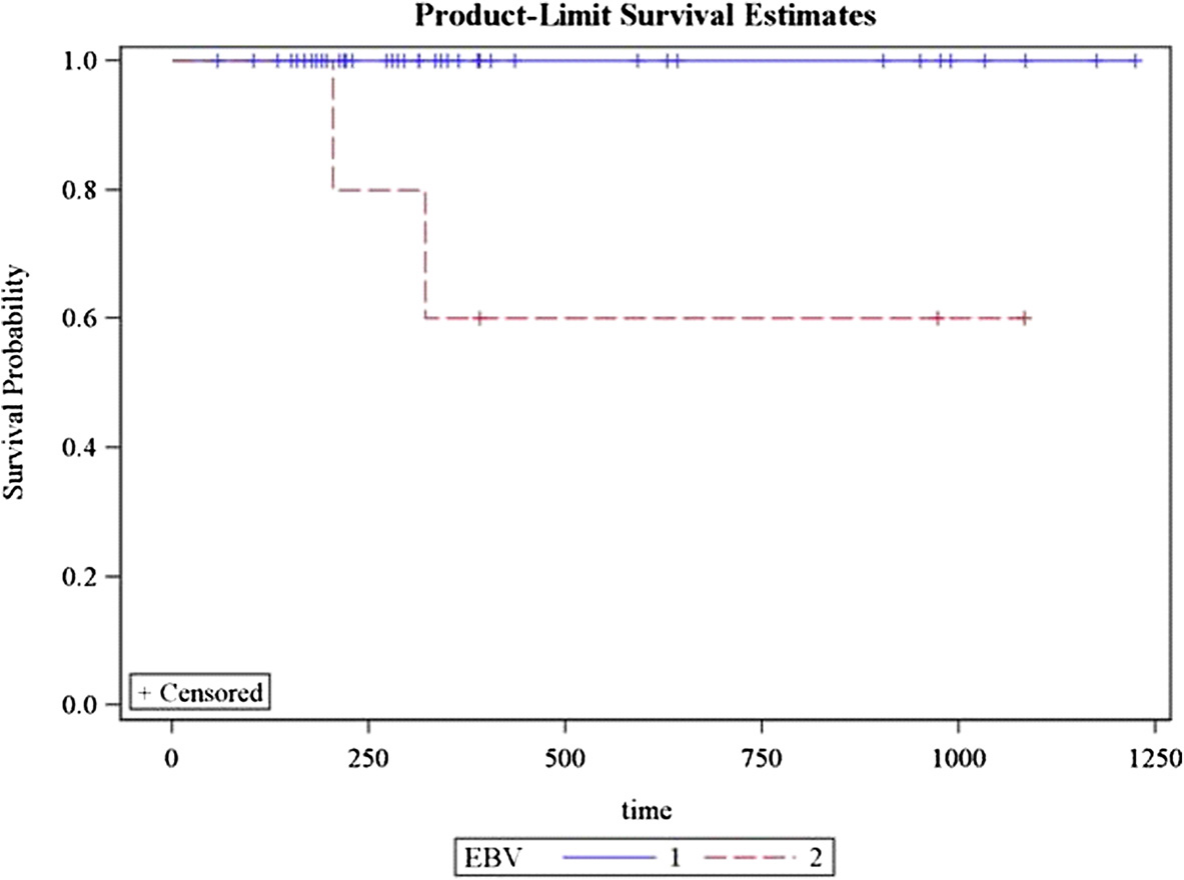
Correlation of Epstein Barr virus with 3 year disease free survival (Blue line shows patients with EBV, Red line indicates those without EBV).

**Figure 3:**
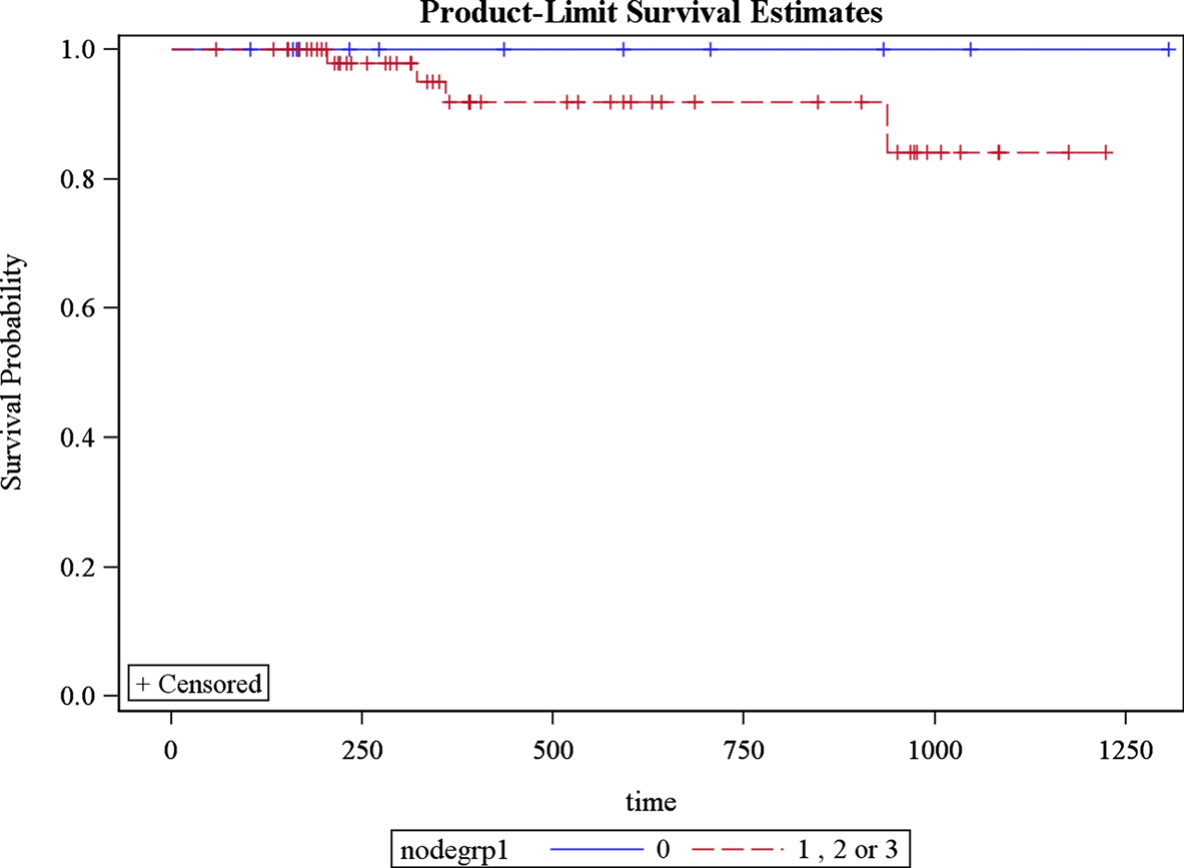
3 year DFS in patients with or without nodal involvement (Blue line indicates patients with N0 disease, whereas red line shows patients having N+ disease).

**Figure 4:**
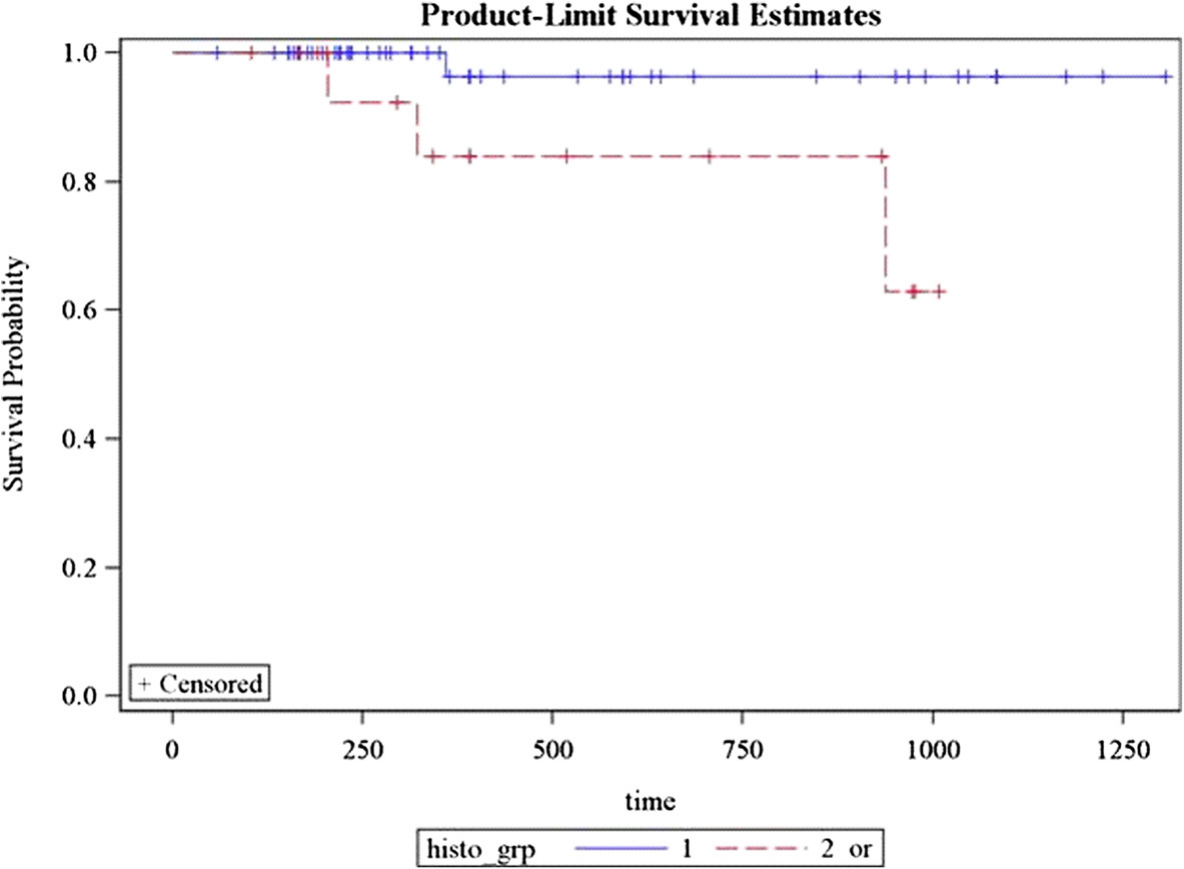
Correlation of histopathology with 3 year DFS (Blue line shows undifferentiated histology, red line indicates all other histologies).

**Figure 5:**
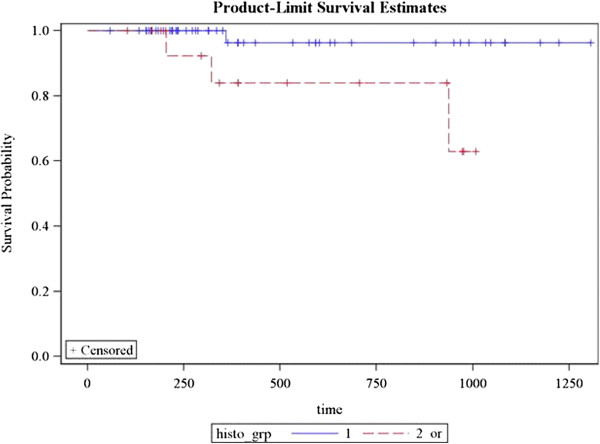
3 Year disease free survival as determined by Kaplan-Meir estimates.

Local recurrence occurred in one patient. Regional recurrence occurred in two patients and distant metastasis in 3 patients. Sixty two patients (91%) achieved complete response while 6 patients had partial response (9%). 3 year DFS for patients with EBV was 100% as compared to 60% without EBV *(p = 0.0009)* (Figure 2)*.* Remission and recurrence rates among N0 patients were 12 (100%) vs. 0%; 47(96%) vs. 2(4%) with N1/N2 and 5 (71.4%) vs. 2 (28.6%) with N3 *(p = 0.02)* (Figure 3)*.* 3 year DFS for patients with undifferentiated histology was 98% as compared to 82% with other histologies *(p = 0.02)* (Figure 4). Estimated 3 year disease free survival (DFS) was 94% (Figure 5).

Univariate and multivariate analysis showed three prognostic factors significantly affected the locoregional control; (a) T stage, (b) N stage and (c) presence of EBV with p values 0.0001, 0.001, 0.002 respectively (Table 2).

**Table 2:** Multivariate analysis of prognostic factors affecting locoregional control in patients with nasopharyngeal carcinoma

**Prognostic factor**	**Hazard ratio**	**95% CI**	***p-*value**
Age groups (years)			
Below 40	1.1	0.8-1.4	0.07
Above 40	1.0	-	0.9
Gender			
Male	1.0	-	0.9
Female	1.0	-	
T stage			
T1 and T2	1.8	1.3-2.2	0.04
T3 and T4	4.2	3.8-4.6	0.0001
N stage			
N0 and N1	0.8	0.6-1.0	0.04
N2 and N3	3.8	3.4-4.2	0.001
EBV status			
Positive	0.7	0.8-1.4	0.002
Negative	1.8	1.3-2.2	0.04

Abbreviations: *T* tumor, *N* node, *EBV* Epstein-Barr virus.

### Acute and late toxicity

Regarding acute toxicity, grade 3 mucositis occurred in 21 patients (30.9%) while 16 patients (23%) and 30 patients (44%) developed grade 1 and grade 2 mucositis. Six patients (8.8%) experienced grade 3 skin reactions whereas 35 patients (51%) had grade 1 and 25 patients (37%) had grade 2 skin reactions. Acute loss of taste took place in 19 patients (27.9%). Late toxicity was minimal with chronic xerostomia (Grade 2) seen in 3 patients (7.5%) among 40 available patients at time of analysis while 10 patients (25%) had Grade 1 Xerostomia. Salivary gland dysfunction occurred in 32 patients (47%), among them 25 (37%) had Grade 1 changes while 7 (10%) had Grade 2 inflammation. After CCRT, Grade 1 impaired hearing occurred in 15 patients (22%) while 3 patients (4.5%) experienced Grade 2 hearing loss.

## Discussion

NPC ranks fourth in the list of first five tumors in Saudi Arabia among young males 30–44 years according to Saudi cancer registry 2005 and about 80% of tumors are T3, T4 or node positive [7]. For such locally advanced NPC patients, chemoradiation incorporating chemotherapy along with IMRT is practical and feasible in terms of clinical efficacy and toxicity. SMART has been employed to further enhance BED in this setting. Long-term results show excellent local tumor control with less late toxicity but no further improvement in overall survival [8]. IMRT with simultaneous integrated boost (SIB) technique for locoregionally advanced NPC was effective regarding locoregional control and development of xerostomia, even after neoadjuvant chemotherapy [9,10]. A review was conducted by Tham et al. on case records of 195 patients with histologically proven, nonmetastatic NPC treated with IMRT between 2002 and 2005. They concluded that local failure or persistent disease, especially in patients with bulky T4 tumors were issues that must be addressed in future trials [11]. In another study by Han L et al., they concluded that IMRT provided favorable locoregional control and survival rates in locally advanced disease. The acute and late toxicities were acceptable and nodal classification was the main factor of prognosis [8]. Ng WT et al. reported 2-year local progression-free, regional progression-free, distant metastasis-free and overall survival rates as 95%, 96%, 90%, and 92%, respectively thus conferring that IMRT provides excellent locoregional control for NPC [12].

This is the first study to evaluate the role of IMRT after induction chemotherapy as well as concomitant with chemotherapy in Saudi Arabia. Overall response was seen in 90% although majority of the patients belonged to stage III & IV (86%) which concludes that IMRT together with chemotherapy, induction and or concomitant is an effective strategy in management of NPC.

One useful biomarker (particularly for nonkeratinizing carcinoma) is the plasma level of EBV deoxyribonucleic acid, and its role as a tool for prognostication and monitoring is rapidly evolving [13]. Our study also proved EBV as a sound prognostic factor.

Historical local control (LC) rates for patients undergoing conventional RT range from 64% to 95% for T1-2 tumors but decreases to 44% to 68% in T3-4 lesions. Most contemporary series have reported encouraging results with CCRT, with locoregional control exceeding 90%; the key problem is distant failure. Although significant reduction of some toxicities (e.g., xerostomia) and better quality of life is now achievable especially for early stages, the risk of major late toxicities remains substantial. Lee et al. showed grade 2 xerostomia in 64% of patients, grade 1 in 28% and grade 0 in 8% at 3 months after IMRT. At 24 months after IMRT, only 1 out of 41 evaluable patients had grade 2, 32% had grade 1 and 66% had grade 0 xerostomia showing that it decreases markedly over time [14]. IMRT enables coverage of irregularly shaped tumor while limiting the dose to critical structures and avoids under dosing the portions of tumors [15].

Neoadjuvant chemotherapy inclusion and radiotherapy optimization methods are two potential areas of interest that need further elaboration in multicenter randomized prospective trials. Phase II studies were done to evaluate the efficacy and safety of neoadjuvant chemotherapy with a regimen of Docetaxel, Cisplatin, and 5 fluorouracil (TPF) followed by radiotherapy and concurrent Cisplatin in patients with stage III and IV (A-B) NPC indicating that it was well tolerated with a manageable toxicity profile [16].

The effect of radiotherapy optimization technique was demonstrated in a recent Italian phase II trial by Palazzi et al. which enrolled 87 patients with NPC who were treated with either conventional (two- or three-dimensional) radiotherapy or with IMRT. Of these patients, 26% received only concurrent cisplatin and the other 74% received both induction and concurrent CT. Three-year DFS and OS were 82% and 90%, respectively. Outcome of NPC further improved in the study period compared with the previous decade, with a significant effect of RT technique optimization [16].

Main limitations of our study are related to its retrospective nature, inability to test all the patients for EBV and non-uniformity of chemotherapeutic agent's selection.

## Conclusions

In conclusion, neoadjuvant CT followed by IMRT and concurrent CT in NPC patients is feasible and effective in terms of toxicity and response. EBV, histopathology and nodal involvement were found to be important prognostic factors for locoregional recurrence.

## Abbreviations

NPC: Nasopharyngeal carcinoma; IMRT: Intensity modulated radiation therapy; SMART: Simultaneous modulated accelerated radiotherapy; CT: Computed tomography; MRI: Magnetic resonance imaging; EBV: Epstein-Barr virus; GTV: Gross tumor volume; CTV: Clinical target volume; PTV: Planning target volume; BED: Biologic effective dose; DFS: Disease free survival; OS: Overall survival; CTC: Common Toxicity Criteria; RTOG: Radiation Therapy Oncology Group; SIB: Simultaneous integrated boost.

## Competing interests

The authors declare that they have no competing interests.

## Authors’ contributions

Concept of Study MMF, ASA, Data Collection RA, Statistical analysis MAT, MM, Manuscript writing MMF, Manuscript editing MAT, YB, Final Review ASI. All authors read and approved the final manuscript.

## Pre-publication history

The pre-publication history for this paper can be accessed here:

http://www.biomedcentral.com/1471-2407/13/318/prepub
